# A deep adversarial model for segmentation-assisted COVID-19 diagnosis using CT images

**DOI:** 10.1186/s13634-022-00842-x

**Published:** 2022-02-10

**Authors:** Hai-yan Yao, Wang-gen Wan, Xiang Li

**Affiliations:** 1 School of Communication and Information Engineering, Shanghai University, Shanghai, China; 2 Anyang Institute of Technology, Anyang, China

**Keywords:** Deep adversarial model, CT image, COVID-19, Deep learning

## Abstract

The outbreak of coronavirus disease 2019 (COVID-19) is spreading rapidly around the world, resulting in a global pandemic. Imaging techniques such as computed tomography (CT) play an essential role in the diagnosis and treatment of the disease since lung infection or pneumonia is a common complication. However, training a deep network to learn how to diagnose COVID-19 rapidly and accurately in CT images and segment the infected regions like a radiologist is challenging. Since the infectious area is difficult to distinguish manually annotation, the segmentation results are time-consuming. To tackle these problems, we propose an efficient method based on a deep adversarial network to segment the infection regions automatically. Then, the predicted segment results can assist the diagnostic network in identifying the COVID-19 samples from the CT images. On the other hand, a radiologist-like segmentation network provides detailed information of the infectious regions by separating areas of ground-glass, consolidation, and pleural effusion, respectively. Our method can accurately predict the COVID-19 infectious probability and provide lesion regions in CT images with limited training data. Additionally, we have established a public dataset for multitask learning. Extensive experiments on diagnosis and segmentation show superior performance over state-of-the-art methods.

## Introduction

The outbreak of coronavirus disease 2019 (COVID-19) is spreading rapidly around the world, resulting in a global pandemic. Timely and accurate detection and diagnosis of the disease are crucial, which would enable the implementation of all the supportive care required by patients affected by COVID-19. Radiological examinations, especially chest computed tomography (CT), play an essential role in the fight against COVID-19. Especially for the timely detection of lung infection abnormalities in the early phase and facilitation of larger public health surveillance and response systems. Since February 13, 2020, chest CT findings have been recommended as the major evidence for confirmed clinical diagnosis, especially in the severely epidemic area. However, ground-glass opacification is often imperceivable on the chest radiography, on which the detection of this type of abnormality is very challenging even for a senior radiologist. Besides, infection annotation by radiologists is a subjective task, since it is often influenced by individual bias and clinical experiences. So artificial intelligence (AI)-based CT imaging models are needed to aid in the fight against the COVID-19 pandemic. The model is expected to yield objective radiologist-like results. It can automatically screen out the infectious areas in lung CT images while simultaneously diagnosing the COVID-19-positive patients from others.

Training a deep network to learn how to diagnose COVID-19 rapidly and accurately in CT images is challenging because the infectious areas are difficult to distinguish. To address these problems, some deep learning-based methods were proposed and utilized in infection detection [1–4], segmentation infection regions [5], classification [6–8], diagnosis [9–11] of COVID-19. Some semi-supervised frameworks [12, 13] have demonstrated good performance due to their high capability of feature extraction. The segmentation methods in COVID-19 application can be mainly grouped into lung regions [8, 14, 15] and lung-lesion regions [5, 16], in terms of the target region of interest (RoI). Since the lesions or nodules could be small with a variety of shapes and textures, locating them is a challenging task. But it is important for further diagnosis.

However, almost all of these methods diagnose by feeding the entire CT image or preprocessing patches of the RoI. There could be two limitations to this strategy. Firstly, only the classification results are given without a proper inference process making the judgment credibility decline. Secondly, the deep model based on the original CT images cannot make full use of the graphical features, leading to false positive and false negative in the data. Thus, we argue that handling this problem using segmentation of the abnormal areas, such as ground-glass, consolidation, and pleural effusion, aiming to assist the classification could be more convincing and effective. Concerned with the COVID-19 radio-graphical changes in CT images, we aim to develop a model that could not only predict COVID-19’s segmentation of the abnormality but also provide a clinical diagnosis ahead of the pathogenic test, thus saving critical time for disease control.

With the impressive progress based on generative adversarial networks (GANs), it is little wonder that unsupervised learning gains considerable attention. Inspired by the successful utilization of GAN in computer vision, we propose a deep adversarial model for segmentation-assisted COVID-19 diagnosis on CT images. Since a standard GAN model [17] has been shown to generate compelling results in a wide variety of applications. Specifically, in the field of medical image segmentation, various GAN-based methods [18–22] have been proposed recently. We try to assist the diagnosis by the accurate radiologist-like segmentation of the infection areas generated by GANs. The reason is that image segmentation is also a marking process, which implies semantic information and detailed features. Moreover, to address the problem of data shortage in the early stage, we try to adopt knowledge distillation [23–26] from imbalanced data.

In this work, our main contributions are twofold:

- First, we employ a GAN-based network to segment the infection areas from the CT image automatically. It is helpful to alleviate the health services by providing a faster way of objectively evaluating the radiological CT images. Efficient radiologist-like segmentation is the key point in further clinical diagnosis.
- Second, we design a segmentation-assisted classification network to diagnose COVID-19. In order to separate the COVID-19 from other diseases, the corresponding annotation can give powerful assistance. Experimental results compared with the state-of-the-art methods show that the proposed method achieves plausible radiologist-like segmentation and accurate diagnosis.

## Related work

Recently, various deep learning approaches [12, 27–29] have been proposed to aid in the fight against the COVID-19 pandemic. We give a brief review of the literature on CT screening, especially approaches compared with the proposed method. For the purpose of this paper, we distinguish two main trends for the related work: segmentation-based methods and classification-based methods.

*Segmentation-based methods* Automatically segmentation of lung infections from CT images offers a great potential to augment the strategy for tackling COVID-19. Lung CT image segmentation is extensively used for COVID-19 detection and diagnosis. Many studies [12, 30–33] exploit segmentation for identifying COVID-19 lung lesions or infection regions. Xue et al. [30] combine U-Net with GAN to achieve the same accuracy and precision as manual annotations when segmenting images. Some researchers [12, 31–33] try to address the class imbalance problem and scarcity of annotation.

*Classification-based methods* Most of the developed COVID-19-diagnosing approaches [34–36] search for specific CT scan patterns and classify them. Tripti et al. [37] utilize a GAN model to classify COVID-19 and non-COVID-19 cases. They optimize the GAN parameters to generate more CT images for diagnosis. Some studies [38–40] prove that VGG network [41], a convolutional neural network (CNN), performs well in terms of accuracy, training convergence, and inference time. Ibrahim et al. [42] introduce a VGG-based method to classify COVID-19, pneumonia, lung cancer, and normal cases. The model shows that the VGG+CNN has better predictive ability.

Different from the methods reviewed above, we propose a deep adversarial model for segmentation-assisted COVID-19 diagnosis to screen CT images. A combination of the segmentation-based and the classification-based method is beneficial for the diagnosis of COVID-19 in the setting of strong clinical suspicion.

## Materials and methods

In this section, we first provide details of the proposed deep adversarial model architecture, including semantic segmentation network and segmentation-assisted diagnosis network. Then, we present the radiologist-like segmentation to annotate different infectious regions. We also show the data distillation technique to address the problem of imbalanced and insufficient data.

### Semantic segmentation network (SSN)

At the very beginning, we think of using a classifier to directly diagnose whether the sample is from COVID-19-positive patients or not. VGG model is selected. We feed the positive and negative samples into a pre-trained model, but the results are not satisfactory. Reasons for the low accuracy are imbalanced training data and high similarity between positive and negative samples. In clinical practice, a radiologist can recognize infection regions and advise on the diagnosis and further treatment. Inspired by this, we aim to train a network to segment like a radiologist and take the segment region as a guide to improve the diagnosis results.

**Fig. 1:**
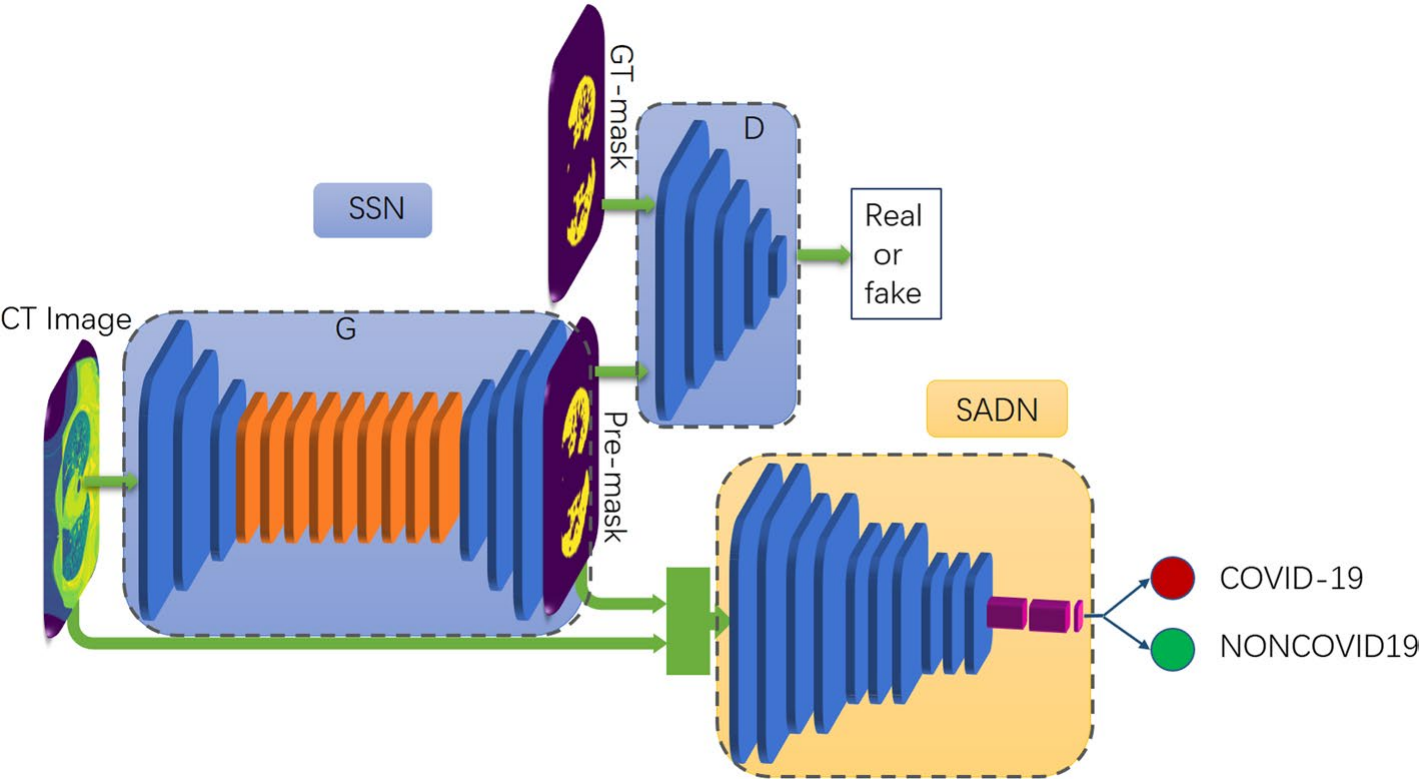
The architecture of the proposed deep adversarial model. The model consists of segmentation network (SSN) and segmentation-assisted diagnosis network (SADN)

In Fig. 1, we provide a schematic representation of the overall framework. The input to our system is CT images waiting for screening and diagnosis. The architecture consists of two main processes: the GAN-based network takes the CT image as input and segments the region of the abnormal area from the background. Furthermore, the predictor takes the CT scan, and the corresponding predicted mask together as input, aiming to diagnose whether it is the COVID-19 or not. In this way, the semantic information can assist the pathogenic test, thus saving critical time and improving accuracy. We present the details of our framework in the following sections.

At the first step of our method, we attempt to employ semantic segmentation network (SSN), a GAN-based network, to force the generated semantic segmentation mask to be more consistent and close to the ground truth. Given image domain *X* and mask domain *M*, the semantic segmentation aims to learn the mapping from a CT scan to a binary mask, $G_{XM}$: $X \rightarrow M$, i.e., segmenting target pixels around abnormality. For generator *G*, it aims to generate a mask that approaches the ground truth. For the discriminator *D*, it can be denoted as $(M, M_{{\mathrm{GT}}})\rightarrow {\mathrm{[real/fake]}}$, distinguishing the true mask $M_{{\mathrm{GT}}}$ from the fake ones *M*. GANs are learned by playing a minimax optimization game between a generator network *G* and a discriminator network *D*. Through this adversarial process, the GAN is capable of learning a generative distribution that matches the empirical distribution of a given data collection. Note that our target is not only generating the mask of abnormality but also aiding the further disease diagnosis. The network requires an accurate segmentation to predict both in location, area, and proportion.

We express the adversarial loss as:

1 $$\begin{aligned} L{_{\mathrm{A}}} = \min \limits _{\theta {\mathrm{g}}} \max \limits _{\theta {\mathrm{d}}} \mathbb {E}{_{x}}[\log (1-D(G(x)))]+ \mathbb {E}{_m}[\log D(m)] \end{aligned}$$

where *x* is the sample of the original CT image, and *m* is the relevant segmentation mask ground truth. $\mathbb {E}$ is the expected value. $\theta _{\mathrm{g}}$ and $\theta _{\mathrm{d}}$ denote the parameters of the network layers of the G and D, respectively. We get these parameters by minimizing the loss of G net and maximizing the loss of D net.

To enhance the efficiency of the segmentation network, a segmentation loss is introduced. In the training process, the mean-squared error (MSE) loss between the generated mask *G*(*x*) (the mapping result of input CT image *x* obtained by the generator *G*) and the ground truth mask $M_{{\mathrm{GT}}}$ of the infectious region. The segmentation loss is defined as follows:

2 $$\begin{aligned} L{_{{\mathrm{seg}}}} = {\mathrm{MSEloss}}(G(x)-M_{{\mathrm{GT}}}) = \sqrt{\frac{1}{N}\sum _{i=1}^N (G(x)-M_{{\mathrm{GT}}})^2} \end{aligned}$$

where *N* is the number of images in the set. The final objective of the SSN is defined as follows:

3 $$\begin{aligned} L{_{{\mathrm{SSN}}}} =L{_{\mathrm{A}}}+\lambda L{_{{\mathrm{seg}}}} \end{aligned}$$

where $\lambda$ is a parameter aiming to keep the balance of the two objectives. We set $\lambda =10$ in training.

The CT images fed to the generator *G* are resized to $512 \times 512$. The generator *G* consists of 24 layers (3 layers for the encoder, 18 layers for the transformer, and 3 layers for the decoder, respectively). In detail, nine residual blocks [50] are used in the transformer, and instance normalization [51] is utilized in the net. Additionally, the discriminator *D* contains five convolution layers. In *G* and *D*, the parameters are 11.37 M and 2.76 M, respectively. We summarize the proposed training procedure for our method in Algorithm 1. All samples are divided into many batches and then fed to the network as one epoch at a time. In the case of limited sample size, more epochs can be run to ensure model convergence. The training process stops when the loss functions no longer descend.

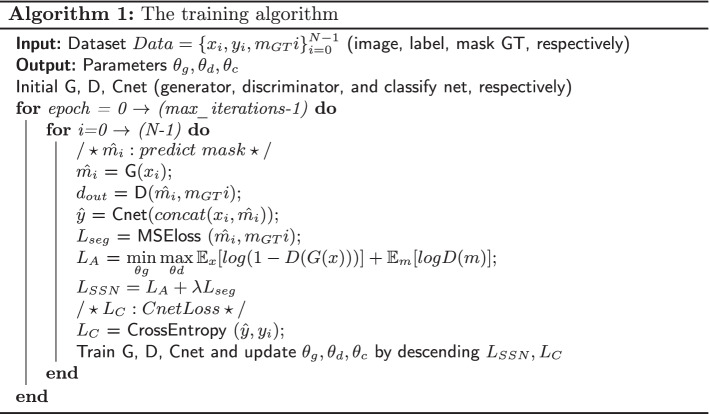



### Segmentation-assisted diagnosis network (SADN)

A classifier is introduced as the segmentation-assisted diagnosis network (SADN). We fine-tune a pre-trained classifier using VGG-16 [41] architecture model. VGG is a classical convolutional network. To provide the semantic information and detailed features to assist the CT slice, we concatenate the generated mask M and the original CT image as the input of the classifier, which is denoted as $C_{{\mathrm{net}}}({\mathrm{concat}}(X, M)) \rightarrow y$, where *y* denotes the label 0/1. The parameters are about 134.27M. In the training process, the CrossEntropy is used as the classification loss.

4 $$\begin{aligned} L{_{\mathrm{C}}} = {\mathrm{CrossEntropy}}(C_{{\mathrm{net}}}({\mathrm{concat}}(x,m))-y) \end{aligned}$$

### Radiologist-like segmentation

**Fig. 2:**
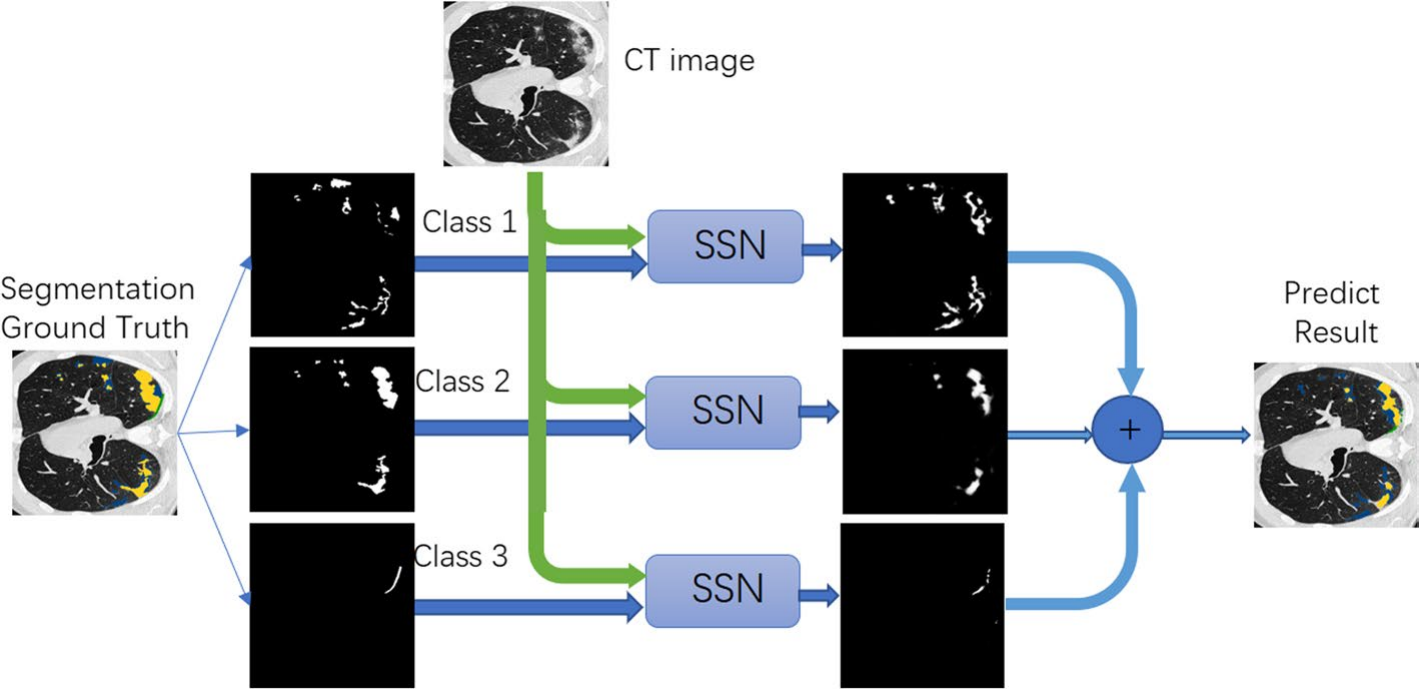
The radiologist-like segmentation (RLS) network has three SSN modules, which segment different infection areas (ground-glass, consolidation, and pleural effusion, respectively). After parallel training, each SSN can separate different classes of the mask. Through compositing the segmentation results into one image, we can achieve the prediction of radiologist-like annotation in infectious areas

Aiming to make the network work as a radiologist and correctly annotate different infection areas, we implement a parallel training strategy. COVID-19 CT segmentation dataset contains images segmented in three labels: ground-glass, consolidation, and pleural effusion with mask values 1, 2, and 3, respectively. We propose the radiologist-like segmentation network (RLS) for the segmentation and diagnosis of multi-sort CT images which is shown in Fig. 2. We feed three types of segmentation ground truth into SSN, accompanied by the original CT image. After parallel training, each SSN can predict different classes of the mask (ground-glass, consolidation, and pleural effusion). The segmentation results of different classes are composited into one image; then, we can achieve the prediction of radiologist-like annotation on infectious areas. The powerful capability of SSN ensures the good performance of RSL, even with imbalanced training data. On chest imaging of COVID-19, multiple small patch shadows and interstitial changes are presented in the early stage, and then, numerous ground-glass shadows are developed in both lungs. In severe cases, lung consolidation could occur, and pleural effusion is rare. We observed very little labeling data for the third category (pleural effusion), leading to training failures. The experimental results show good predictions in the good segmentation and classification. This detailed, radiologist-like segmentation can give clinicians great help in determining the extreme severity of the disease.

### Data distillation for COVID-19 diagnosis

We introduce data distillation to the deep adversarial network. To address the problem of insufficient data, we leverage data distillation on unlabeled data. Firstly, we train a teacher model on manually labeled data. Secondly, the trained model is applied for segmentation. Then, we convert the predictions on the unlabeled data into labels by assembling the multiple predictions. Finally, the model is retrained on a new training set consisting of manually labeled data and automatically labeled data. The aim is to mine latent information in the data at hand, including limited labeled samples and more unlabeled samples. It is helpful to improve the performance of the network.

## Experimental results and discussions

### Datasets and metrics

*Datasets* As we can see in Table 1, existing public datasets are either for diagnosis or for segmentation tasks. The COVID-19 CT segmentation dataset [43] is the first one for segmentation, containing 100 axial CT images from 60 patients with COVID-19. Each image is segmented by a radiologist using three labels: ground-glass (mask value = 1), consolidation (mask value = 2), and pleural effusion (mask value = 3). Then, COVID-SemiSeg dataset [12] adds 1600 unlabeled images from the COVID-19 CT Collection dataset [44] for augmenting the training data. It is helpful for training a deep network to diagnose like a radiologist.

**Table 1:** Comparison of the open-source dataset sources of COVID-19 imaging. (Diag means diagnosis and Seg means segmentation)

Dataset	Data type	Size (Cov/NonCov)	Task-Diag	Task-Seg
COVID-19 X-ray collection [44]	X-rays	229	$\checkmark$	–
COVID-19 CT collection [44]	CT volume	20	$\checkmark$	–
COVID-CT-dataset [45]	CT image	349/1000	$\checkmark$	–
COVID-19 patient lungs [46]	X-rays	70/28	$\checkmark$	–
COVID-19 radiography [47]	X-rays	1143/1314/1345 (viral pneumonia)	$\checkmark$	–
SARS-CoV-2 CT-scan [48]	CT image	1252/1230	$\checkmark$	–
COVID-19 CT segmentation [43]	CT image	110/0	–	$\checkmark$
COVID-SemiSeg [12]	CT image	1700/0	–	$\checkmark$
COVID-19 CT lung and infection segmentation [49]	CT image	20/0	–	$\checkmark$
COVID-19 radiologist dataset	CT image	100/93	$\checkmark$	$\checkmark$

Inspired by it, we collect a COVID-19 radiologist dataset for multitask learning. For the segmentation task, we employ our network on the 100 images with segment labels from the COVID-19 CT segmentation dataset [43]. The images are resized, grey-scaled, and compiled into a single NIFTI-file (512 $\times$ 512 $\times$ 110). We split the 100 images into five equal folds, using 4 of them for training and 1 for testing. Then, we iterate over which fold is the test fold, evaluate the performance, and finally average the performance across the different folds. For the diagnosis task, the training set consists of 100 COVID images used in the segmentation task and another 100 non-COVID images from COVID-CT-Dataset [45]. For testing, we collect another 275 labeled COVID images and 818 labeled non-COVID images from COVID-CT-Dataset. In this way, we implement our deep adversarial network and verify that the segmentation results can improve the diagnosis performance. The experimental results demonstrate the effectiveness of the proposed approach.

*Metrics* For the diagnosis task, the performance of the model was evaluated by assessing the classification accuracy, precision, recall, and F1 scores. The accuracy of a method determines how correct the values are predicted. The precision determines the reproducibility of the measurement or how many of the predictions are correct. Recall shows how many of the correct results are discovered. F1 score uses a combination of precision and recalls to calculate a balanced average result. For the segmentation task, we evaluate the results by pixel accuracy (PA) and intersection over union (IOU). PA is the simplest metric defined as the ratio of the marking correct pixels to the total pixels. IOU is a natural criterion for semantic segmentation.

### Segmentation results and analysis

The segmentation results can visually see the infectious areas, which is more beneficial for clinicians to diagnose the disease. So we implement the typical infection segmentation firstly. Then, aiming to show the detailed result, we train the network to segment like a radiologist.

**Fig. 3:**
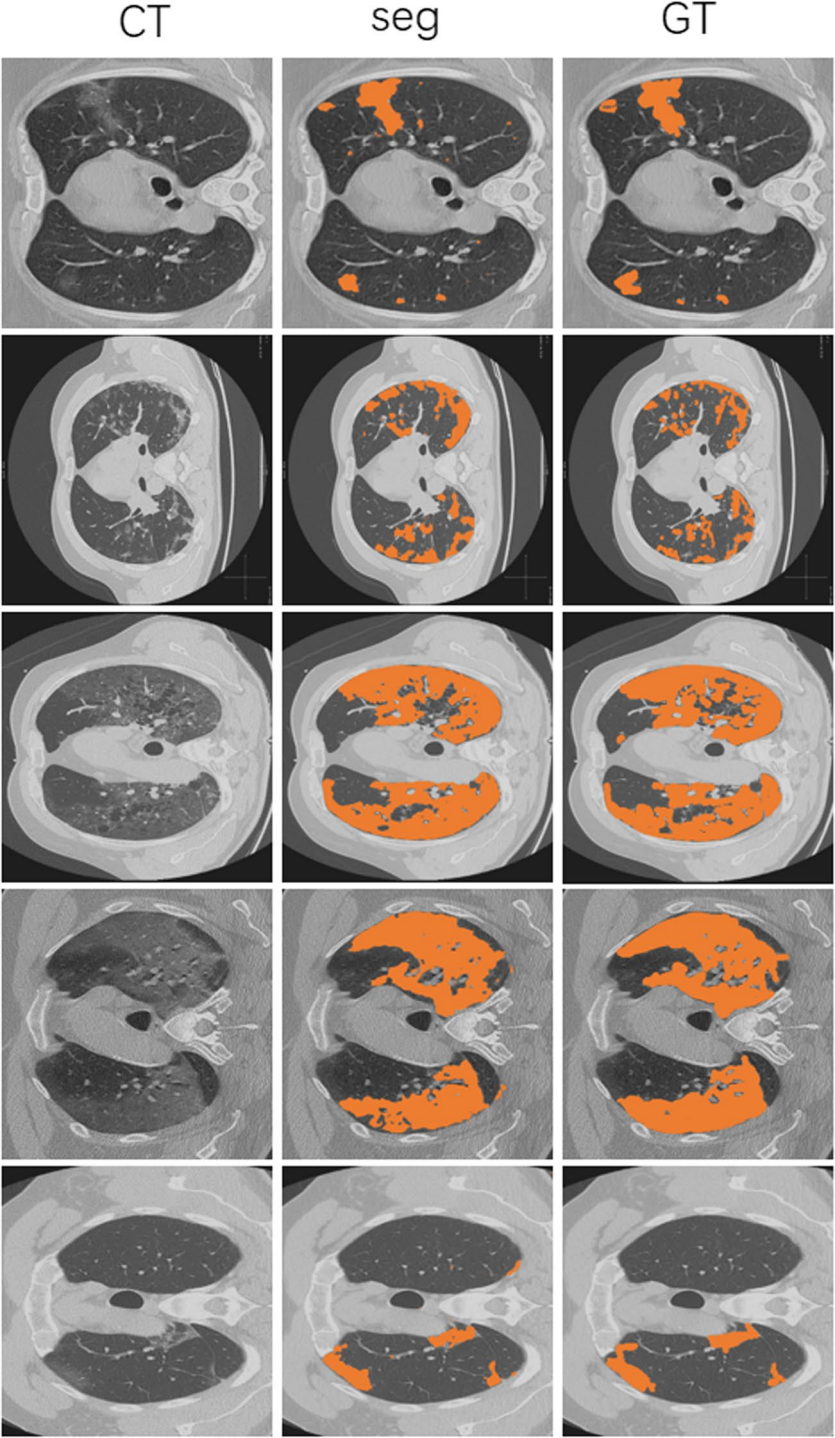
Typical infection segmentation (TIS) results of infectious areas. We feed the slices to SSN and train it to segment the infection region. The TIS results show high-quality segmentation masks for each pulmonary infection area. Columns 1–3: CT image, CT image overlaid with segmentation, and ground truth, respectively

*Typical infection segmentation (TIS)* We feed the slices to SSN and train it to segment the infection region. In Fig. 3, we can see the typical infection segmentation (TIS) results of SSN, from which we achieve high-quality segmentation masks for each pulmonary infection area. Except for the small parts on the boundary that are error-prone, as shown in the annotated section in the bottom row. This may be due to the network’s inadequate identification of small and fragmented targets, but it does not affect the estimation of COVID-19. We can achieve infection segmentation and COVID-19 classification at the same time.

The comparison results of different segmentation methods are shown in Table 2; as we can see, the proposed TIS can get higher performance than Mask-RCNN [52] (a classical segmentation method). Specifically, the PA and IOU increase by 0.09 and 13.28 percent, respectively. The best results compared with other methods are in boldface.

**Table 2:** Typical infection segmentation (TIS) results comparison of different methods on COVID-19 radiologist dataset

Method	PA	IOU
Mask-RCNN [52]	84.96%	44.8%
TIS	**85.05%**	**58.08%**

**Fig. 4:**
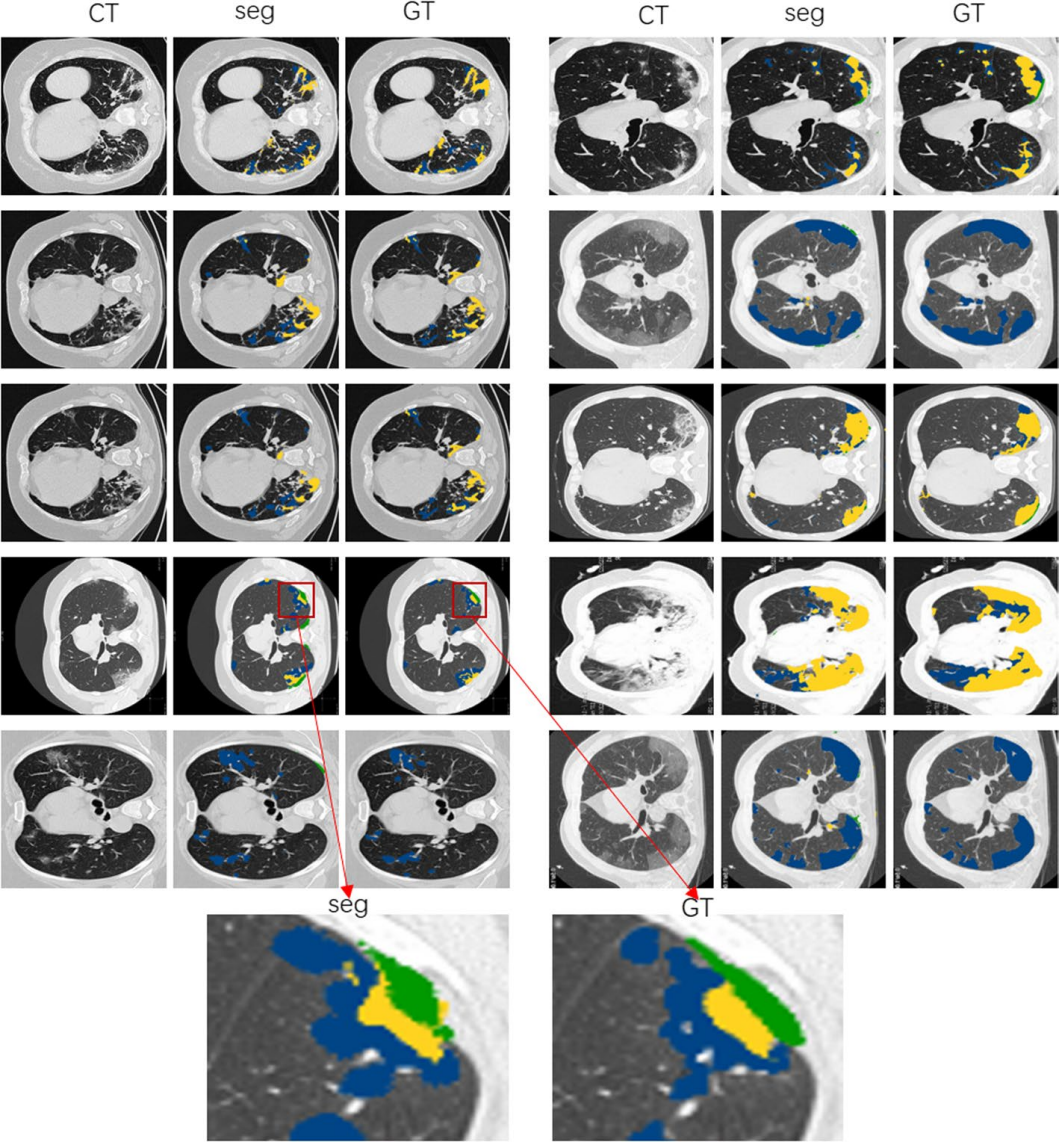
Radiologist-like segmentation (RLS) results of infectious areas in different classes. Columns 1–3: CT image, CT image overlaid with segmentation, and ground truth. The blue, yellow, and green labels indicate the ground-glass, consolidation, and pleural effusion, respectively. The partial enlarged view shows the high reliability of the network. It can generate high-quality segmentation results even in complex regions with different classes

*Radiologist-like segmentation (RLS)* Furthermore, to make the network work as a radiologist, we train the SSN in a parallel strategy. The refined segmentation results, shown in Fig. 4, indicate that our method can yield masks similar to manual annotation. The blue part, yellow part, and green part represent ground-glass, consolidation, and pleural effusion, respectively. Compared to the ground truth segmentation annotated by a real radiologist, the network can provide plausible results, especially ground-glass (blue) and consolidation area (yellow). The partial enlarged view shows the high reliability of the network. It can generate high-quality segmentation results even in complex regions with different classes.

**Fig. 5:**
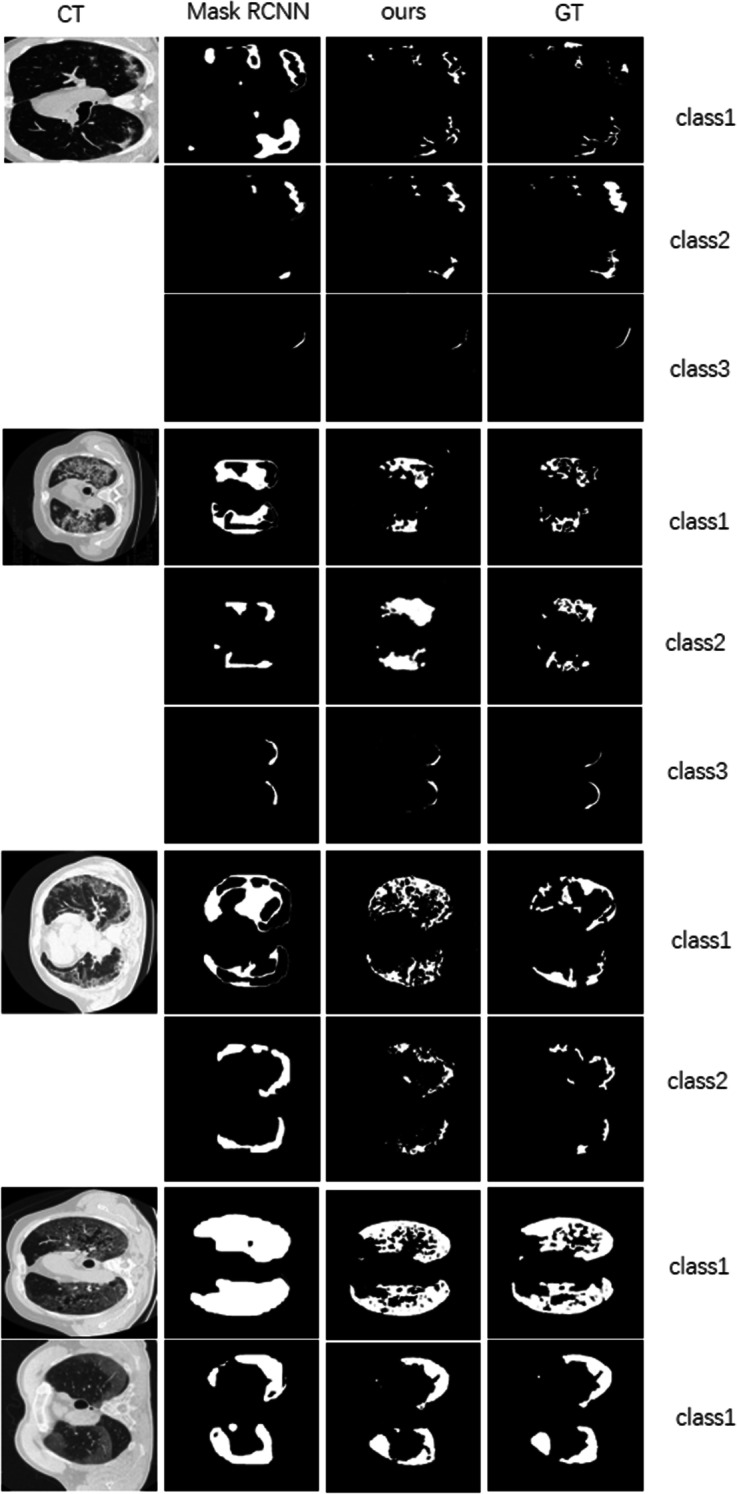
Visual comparison of infection segmentation in detail. Columns 1–4: CT image, segmentation results by Mask-RCNN, segmentation results by our method, and ground truth. Mask-RCNN has a good segmentation effect on coherent graphic areas. But it joins small incoherent areas together, resulting in detail lost phenomena. The proposed method makes detailed detection and gets good segmentation results in terms of incoherent region

Radiologist-like segmentation (RLS) is more challenging than typical infection segmentation (TIS) because it requires not only segmentation but also classification of the results. Therefore, it is prone to confuse when discriminating different classes of infection regions. Take the result on the right of the fourth row in Fig. 4 as an example; consolidation is misjudged as ground glass (i.e., misjudge the blue part as yellow).

The performance of the RLS method with different loss functions is listed in Table 3. Firstly, we train the SSN and calculate the L1 loss between the real mask and the generated mask; class 1 and class 2 segment results are acceptable. However, the segmented effect of class 3 is not obvious (PA and IOU are 1% and 0.27%, respectively). So, we change to use MSE loss for training; the segmentation results on the three classes are improved. Specifically, the results on class 1 and class 2 are enhanced remarkably, which can be seen in Table 3. This indicates that MSE loss is more beneficial to the network than L1 loss. In addition, we compare the result with Mask-RCNN, which is good at the segmentation task. Mask-RCNN gets a slightly better PA (77.08%) than the RLS with MSE loss (76.02%) in class 1 segmentation task. Beyond that, RLS performs better scores in other ways, which can be observed in Table 3. The best results are in boldface. The comparison segmentation results with different methods, shown in Fig. 5, indicate that our method outperforms Mask-RCNN remarkably. We show samples of three different cases: samples with three classes, only 1 and 2 classes, and only 1 class. We observe that mask-RCNN has a good segmentation effect on coherent areas. But it joins small incoherent areas together, resulting in detail lost phenomena. The proposed method makes detailed detection and gets good segmentation results in terms of incoherent regions. Therefore, we achieve better performance in PA and IOU.

**Table 3:** Radiologist-like segmentation (RLS) results comparison of different methods on COVID-19 radiologist dataset

Method	Class	PA	IOU
Mask-RCNN [52]	1	**77.08%**	31.42%
RLS with L1loss	1	66.43%	46.93%
RLS with MSEloss	1	76.02%	**48.15%**
Mask-RCNN [52]	2	46.74%	24.66%
RLS with L1loss	2	42.42%	26.82%
RLS with MSEloss	2	**62.82%**	**32.10%**
Mask-RCNN [52]	3	26.35%	13.62%
RLS with L1loss	3	2.33%	0.27%
RLS with MSEloss	3	**30.48%**	**18.12%**

Compared with others, class 3 segmentation is more challenging. The reason is when the segmentation process targets rare observations, a severe class imbalance is likely to occur between candidate labels. A parallel strategy is utilized in this case. To increase the proportion of class 3 samples in training, we split the train set into class 3 and non-class 3 and randomly selected samples in each batch. Rotation, flip, and crop are also used for data augmentation.

### COVID-19 diagnosis results

We show the effect of mask assistant prediction. We train and test our network on the COVID-19 radiologist dataset and achieve a good result. Considering that the classification does not occur simultaneously with segmentation, we select a more robust model after segmentation to assist the classification procedure (with mask). Compared with the direct classification (without mask), we can see that the estimated mask from SSN can significantly improve the classification result. In addition, the classification results with Mask-RCNN segment assistant are shown as a comparison in Table 4, which is a little bit lower than the proposed method. The best results are in boldface.

**Table 4:** Result comparison of different diagnosis methods on COVID-19 radiologist dataset: classifier with mask, only use classifier without SSN (W/O mask), and with SSN assistant classification (proposed), respectively

Method	Accuracy	Precision	Recall	F1
With mask	96.04%	90.88%	94.12%	93.5%
W/O mask	95.06%	87.97%	93.09%	90.46%
Proposed	**99.2%**	**98%**	**96.0%**	**97.96%**

### Data distillation results

**Fig. 6:**
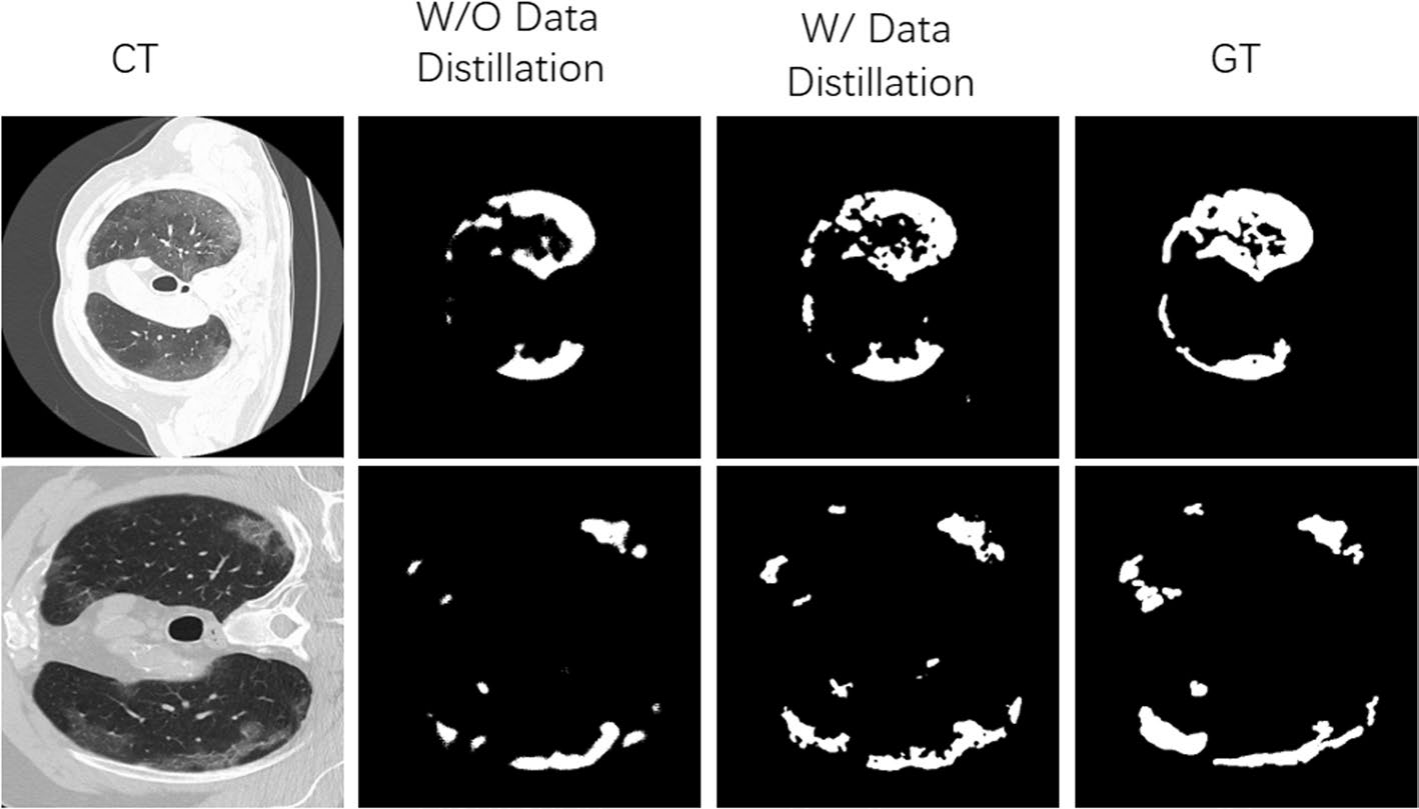
Results comparison of segmentation with and without data distillation. The data distillation can enhance the segmentation performance obviously. Segmentation results of ground-glass have obvious edges, and clearly discrimination of the target area and non-target area after data distillation. Columns 1–4: CT images, segmentation results W/O data distillation, segmentation results with data distillation and ground truth

Data distillation can boost the performance of segment results, as shown in Fig. 6. We exploit a comparison experiment on radiologist-like segmentation to illustrate the effect of data distillation. Figure 6 shows that segmentation results of ground-glass have obvious edges, and clear discrimination of the target area and non-target area after data distillation. The reason is to mine the information in the existing labeled data. For unlabeled data, we also make full use of the hidden information for further prediction. Yet it has limits. The data distillation has been proved capable of tackling the problem of the insufficient data problem by utilizing more unlabeled samples, but incapable of keeping the certainty in training since introducing error-prone labels, so-called pseudo labels, as ground truth.

### Discussion

As our experimental results showed, we achieve promising results in COVID-19 diagnosis, and segmentation of the infection regions such as ground-glass, consolidation, and pleural effusion regions. These results are helpful for clinicians to make a rapid diagnosis and treatment plans. There are two advantages to the deep adversarial model. Firstly, we conduct a cascade strategy to achieve segmentation and diagnosis. The results generated by SSN serve as the auxiliary of the original CT image, thus improving the results of the diagnosis. Secondly, it is simple to use, and no complicated techniques are introduced. The results of diagnosis and segmentation are mutually promoted. Moreover, we can clearly see or estimate the performance of each step.

Even though there are some limitations. Firstly, when we conduct a multistep strategy to solve a complex task, it is obvious that each step’s training objective is not consistent. How to balance the relation and train the network to achieve optimal performance is a challenging because the deviation from one step may affect another. In the future, we will focus on constructing an end-to-end network to tackle this problem. Second is the problem of efficient learning on imbalanced data. Annotation data are challenging since it needs an experienced radiologist. And manually labeling different kinds of pulmonary infection areas with different labels is time-consuming. Even though we use a parallel strategy, segmenting the very small sample size in one class is still a challenge. We will study to optimize the model to make full use of the existing samples in the future.

## Conclusion

In this work, we construct a deep adversarial network that can predict radiologist-like segmentation of the lung’s infectious region and provide a clinical COVID-19 diagnosis ahead of the pathogenic test, thus saving critical time for disease control. The segmentation results bring detailed and important information to assist the classifier. This information effectively improves the COVID-19 diagnosis accuracy. Experiments show successful results on both of the tasks. Moreover, a parallel training strategy and data distillation technique are utilized to tackle the problem of imbalanced and insufficient data in the training process, which can enhance the network performance.

## Data Availability

All the data are available upon request from the corresponding author.

## Abbreviations

COVID-19: Coronavirus disease of 2019
CT: Computed tomography
CXR: Chest X-ray
RoI: Region of interest
GAN: Generative adversarial network
VGG: Visual geometry group
SSN: Semantic segmentation network
RSN: Radiologist-like segmentation network
TIS: Typical infection segmentation
